# O-GlcNAcylation-Inducing Treatments Inhibit Estrogen Receptor α Expression and Confer Resistance to 4-OH-Tamoxifen in Human Breast Cancer-Derived MCF-7 Cells

**DOI:** 10.1371/journal.pone.0069150

**Published:** 2013-07-11

**Authors:** Shahzina Kanwal, Yann Fardini, Patrick Pagesy, Thierry N’Tumba-Byn, Cécile Pierre-Eugène, Elodie Masson, Cornelia Hampe, Tarik Issad

**Affiliations:** 1 Institut Cochin, Université Paris Descartes, CNRS (UMR8104), Paris, France; 2 INSERM, U1016, Paris, France; King's College London, United Kingdom

## Abstract

O-GlcNAcylation (addition of N-acetyl-glucosamine on serine or threonine residues) is a post-translational modification that regulates stability, activity or localization of cytosolic and nuclear proteins. O-linked N-acetylgluocosmaine transferase (OGT) uses UDP-GlcNAc, produced in the hexosamine biosynthetic pathway to O-GlcNacylate proteins. Removal of O-GlcNAc from proteins is catalyzed by the β-N-Acetylglucosaminidase (OGA). Recent evidences suggest that O-GlcNAcylation may affect the growth of cancer cells. However, the consequences of O-GlcNAcylation on anti-cancer therapy have not been evaluated. In this work, we studied the effects of O-GlcNAcylation on tamoxifen-induced cell death in the breast cancer-derived MCF-7 cells. Treatments that increase O-GlcNAcylation (PUGNAc and/or glucosoamine) protected MCF-7 cells from death induced by tamoxifen. In contrast, inhibition of OGT expression by siRNA potentiated the effect of tamoxifen on cell death. Since the PI-3 kinase/Akt pathway is a major regulator of cell survival, we used BRET to evaluate the effect of PUGNAc+glucosamine on PIP_3_ production. We observed that these treatments stimulated PIP_3_ production in MCF-7 cells. This effect was associated with an increase in Akt phosphorylation. However, the PI-3 kinase inhibitor LY294002, which abolished the effect of PUGNAc+glucosamine on Akt phosphorylation, did not impair the protective effects of PUGNAc+glucosamine against tamoxifen-induced cell death. These results suggest that the protective effects of O-GlcNAcylation are independent of the PI-3 kinase/Akt pathway. As tamoxifen sensitivity depends on the estrogen receptor (ERα) expression level, we evaluated the effect of PUGNAc+glucosamine on the expression of this receptor. We observed that O-GlcNAcylation-inducing treatment significantly reduced the expression of ERα mRNA and protein, suggesting a potential mechanism for the decreased tamoxifen sensitivity induced by these treatments. Therefore, our results suggest that inhibition of O-GlcNAcylation may constitute an interesting approach to improve the sensitivity of breast cancer to anti-estrogen therapy.

## Introduction

Growth and proliferation of cancer cells tightly depend on their nutritional environment, particularly on glucose availability, which is necessary for increased biosynthesis of cellular components associated with proliferation (e.g. membranes, proteins and nucleic acids) [1]. Nutritional and metabolic conditions are known to influence tumour development. Excess food intake associated with modern lifestyle constitutes an important cancer risk factor [2]. In animals, food restriction has inhibitory effects on the growth of certain tumours [3], whereas in diet-induced obesity models, overfeeding is associated with accelerated development of tumours [4].

**Figure 1:**
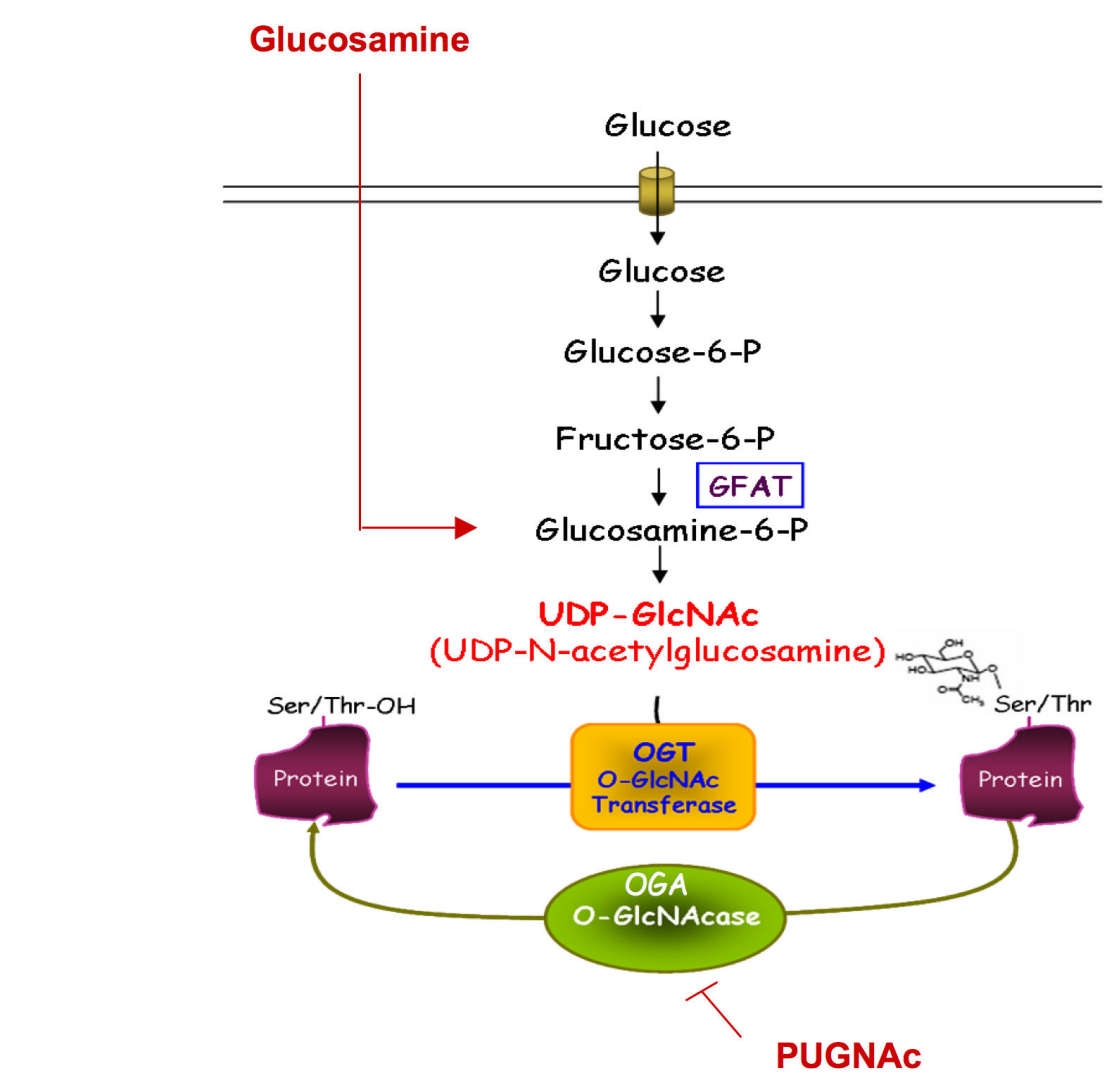
The hexosamine biosynthetic pathway controls O-GlcNAc-modification of proteins. Cytosolic and nuclear O-GlcNAc glycosylation constitutes a dynamic process that regulates the activity, the localisation or the stability of the modified proteins. O-GlcNAc glycosylation of proteins on serine and threonine residues depends on the flux of glucose through the hexosamine biosynthetic pathway (HBP). A fraction (2 to 5%) of the glucose entering the cell is directed into this pathway for the biosynthesis of UDP-GlcNAc. OGT uses UDP-GlcNAc as a substrate to add GlcNAc on serine or threonine residues, and its activity is tightly dependent on the concentration of UDP-GlcNAc in the cell. These modifications can be reversed by O-GlcNAcase (OGA), which removes the O-GlcNAc moiety from O-GlcNAc-modified proteins. Experimentally, the level of O-GlcNAc in proteins can be increased by incubating cells with glucosamine (which bypasses allosteric inhibition of the rate limiting enzyme GFAT (Glutamine Fructose 6-P amidotransferase)), or with PUGNAc (O-[2-acetamido-2-deoxy-D-glucopyranosylidene] amino-N-phenylcarbamate), which inhibits deglycosylation by OGA.

Nutritional conditions can modulate tumour development by modifying insulin and IGF-1 concentrations, which affect signalling pathways involved in cell growth, proliferation and apoptosis. However, at the cellular level, glucose can also directly regulate signalling pathways and multiple biological processes through O-GlcNAc glycosylation (O-GlcNAcylation) of cytosolic and nuclear proteins [5]. O-GlcNAcylation is a reversible post-translational modification, analogous to phosphorylation, which controls protein localisation, stability or activity according to the nutritional environment. It corresponds to the addition of N-Acetylglucosamine (GlcNAc) on serine or threonine residues. This reaction is catalysed by O-GlcNAc transferase (OGT), which uses UDP-GlcNAc as a substrate (Figure 1). UDP-GlcNAc, produced through the hexosamine biosynthetic pathway (HBP), can be considered as a sensor for the nutritional state of the cell, as it integrates glucose, glutamine, fatty acids (acetyl), uridine and ATP metabolism [5–9]. O-GlcNAc is rapidly removed from proteins by O-GlcNAcase (OGA), permitting dynamic regulation of O-GlcNAcylation levels in cells.

A growing amount of studies indicates that O-GlcNAcylation constitutes an important regulator of cancer growth and invasion. A number of transcription factors involved in the control of cell proliferation or cell death can be regulated by O-GlcNAc [10–15]. Moreover, increased protein O-GlcNAcylation has been detected in cells derived from breast, lung, colon, liver and prostate cancers. These modifications, often associated with changes in OGT and/or OGA levels [16–18], favour the growth and/or migration of tumour cells through different mechanisms, including regulation of transcription factors activity [19,20], E-cadherin [18,21], and MMP metalloproteases [18,20].

Breast cancer is the most common cancer in women. Endocrine therapies have permitted important progress for the treatment of hormone-sensitive breast cancers. However, the development of treatment resistance constitutes an important limitation to these therapies. Thus, tamoxifen, a partial antagonist of the estrogen receptor, has been largely used for the treatment of estrogen receptor positive breast cancers, but resistance to this drug often occurs [22]. The link between nutritional conditions, obesity and breast cancer is well established, particularly in menopausal women [23]. However, few studies have evaluated the consequences of metabolic manipulations on the efficiency of anti-cancer therapy. In this work, we studied the effect of treatments that induce protein O-GlcNAcylation on the sensitivity to tamoxifen, using the human breast cancer derived, estrogen receptor positive MCF-7 cell line.

## Methods

### Chemicals and antibodies

Glucosamine (GlcN) was from Sigma-Aldrich, O-(2-acetamido-2-deoxy-D-glucopyranosylidene)-amino-N-phenylcarbamate (PUGNAc) from Toronto Research Chemicals Inc., and LY294002 from Cell Signaling Technology. Anti-Akt, anti-Phospho-Akt and anti-phospho-Erk1/2 antibodies were from Cell Signaling Technology, anti-ERα (60C) from Millipore, anti-O-GlcNAc (CTD 110.6) from Covance, anti-GAPDH from Life Technologies, anti-β-actin from Sigma, anti-Erk2 (sc 154) and HRP-conjugated donkey anti-rabbit (sc 2305) from Santa Cruz, and HRP-conjugated goat anti-mouse from Jackson ImmunoResearch laboratories.

### Cell culture and transfection

MCF-7 cells were maintained in DMEM supplemented with 10% fetal bovine serum. Transfection of siRNA and cDNAs were performed with Lipofectamine RNAi Max (Life Technologies) and FuGene 6 (Promega), respectively. The negative control siRNA was from Eurogentec (Seraing, Belgium).

### Uptiblue assay

Cells were plated in clear 96-well-plates at a density of 2000 cells/well. 24 h after plating, cell population density was evaluated 2h30 after adding 10% Uptiblue reagent, by measuring fluorescence (initial fluorescence) at 595 nm using Typhoon 9400 imager (GE Healthcare). Cells were then cultured for 24 or 48 h in fresh media containing the different agents, and then the final fluorescence was measured. The cell population growth in each well was expressed as the ratio of the final fluorescence over the initial one in the same culture well [24].

### Apoptosis analysis

After treatment in 6-well-plates, cells were harvested by trypsin digestion, washed in PBS and labelled with both Annexin-V-FITC and propidium iodide using the Annexin-V FLUOS kit (Roche Diagnostics) according to manufacturers’ instructions. Cells were analyzed by flow cytometry with the FC 500 cytometer (Beckman Coulter).

### BRET experiments

Luc-Akt-PH and YFP-Mem cDNAs used for the study of PIP_3_ production have been previously described [25]. MCF-7 cells were transfected with 0.7 µg Luc-Akt-PH and 0.3 µg pYFP-Mem cDNAs per 10.3 mm dish and transferred to a 96 well plate 24 h before BRET experiments. BRET experiments were performed as described previously [26,27]. Results were expressed in milliBRET units as previously described [28,29].

### Luciferase assay

The *ESR1-Luc* reporter gene (firefly luciferase coding sequence under the control of the P1 promoter of *ESR1* gene [30], kindly provided by Dr. M. LE ROMANCER-CHERIFI) was used. Cells were seeded in 12-well-plates and transfected 24 h later with 1 µg of ESR1-Luc plasmid combined with 2 ng of a Renilla luciferase cDNA to normalize for transfection efficiency. After treatment, cells were lysed and luciferase activities were measured with a Centro LB 910 luminometer (Berthold) using the DUAL Luciferase Assay kit (Promega).

### Western-blotting

MCF-7 cells were lysed with buffer containing 50 mM Tris–HCl (pH 8), 137 mM NaCl, 10% (v/v) glycerol, 1% (v/v) NP40, 50 mM NaF, 10 mM di-sodium β-glycerophosphate, 1 mM Na _3_VO_4_, 1 mM streptozotocin and protease inhibitors (1µg/ml pepstatin, antipain, leupeptin, aprotinin and AEBSF). Proteins were then analysed by SDS-PAGE followed by western-blotting [31].

### RNA extraction, Reverse Transcription and qPCR

After treatment, cells cultured in 6-well-plate were lysed directly in Trizol reagent (Life Technologies) and RNA were isolated and reverse transcribed as previously described [32]. Levels of the cDNA of interest were measured by qPCR using LightCycler FastStart DNA Master SYBR Green 1 kit. To ensure absence of genomic DNA contamination, RNA samples were treated in parallel without Reverse Transcriptase and controlled for absence of amplification by qPCR. The sequences of the primers used in these experiments are: ESR1: Forward, GCATTCTACAGGCCAAATTCAG, Reverse, GTCGTTATGTCCTTG AATACTTC; p21: Forward, TGTACCCTTGTGCCTCGCTCAG; Reverse, TGTA CCCTTGTGCCTCGCTCAG; EGR1: Forward, GCACCTGACCGCAGAGTCTT, Reverse, AGTGGTTTGGCTGGGGTAACT; Cyclophilin A: Forward, GGTGACTTC ACACGCCATAATG, Reverse, ACAAGATGCCAGGACCCGTAT. Gene expression was normalized using cyclophilin A mRNA level as a reference.

### Statistical analysis

Statistical analyses were performed with Prism software (GraphPad) using either a *t* test or ANOVA followed by post-test as indicated in figure legends.

## Results

### O-GlcNAcylation-inducing treatments protect MCF-7 cells from 4OH-Tamoxifen-induced cell death

We used the Uptiblue, which monitors the cellular growth within the same well, to evaluate the effect of different treatments on MCF-7 cells [24]. Cells were treated with PUGNAc (inhibitor of OGA) and glucosamine (bypasses the GFAT rate limiting step) in absence or presence of 4-OH-Tamoxifen. Treatment with PUGNAc and glucosamine (GlcN) markedly increase O-GlcNAcylation of proteins in MCF-7 cells, both in the absence and presence of 4-OH-Tamoxifen (Figure S1).

**Figure 2:**
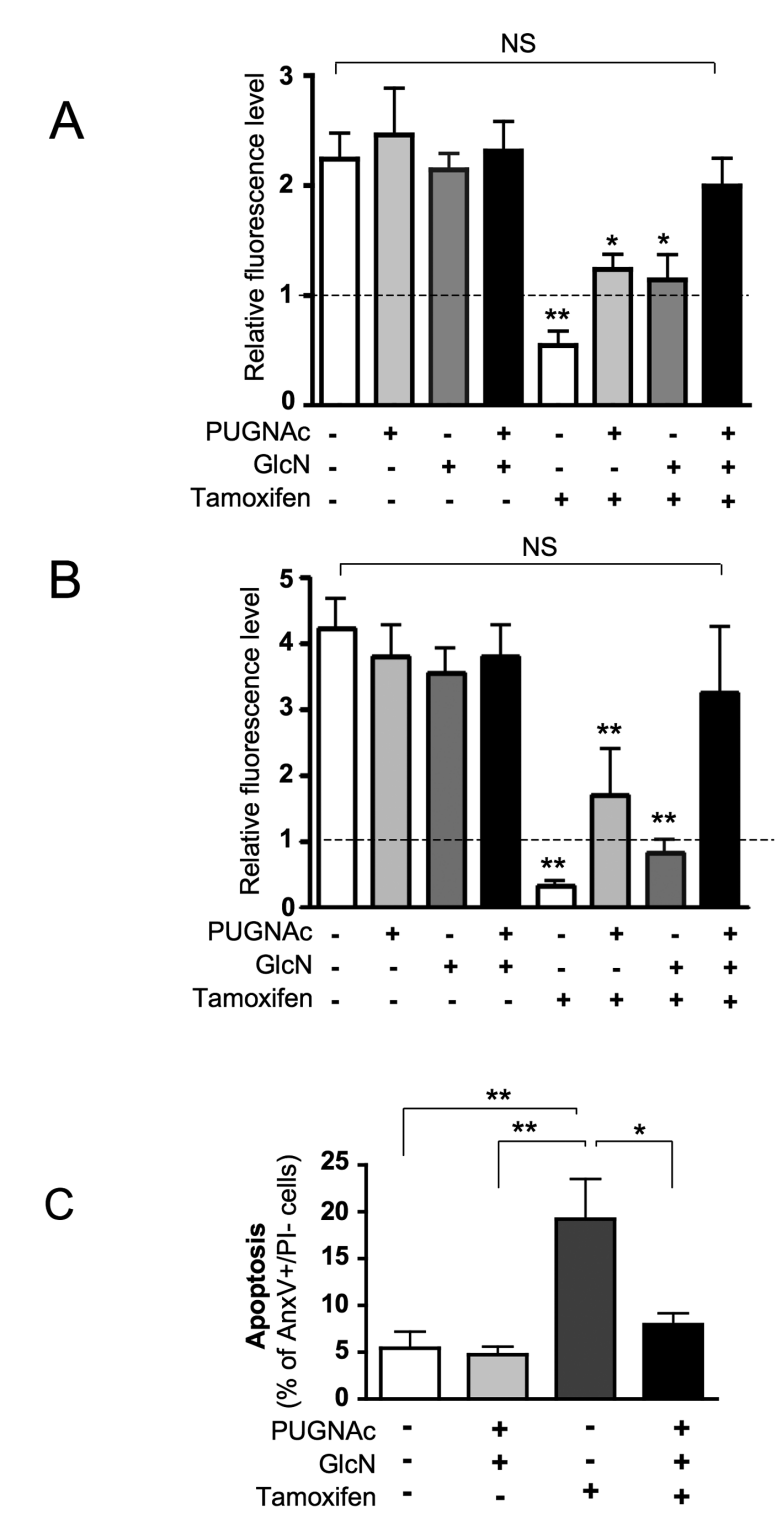
O-GlcNAcylation-inducing treatments protect from tamoxifen-induced cell death. MCF-7 cells were cultured in presence of 1% FBS during 24 h (A) or 48 h (B), in the absence or presence PUGNAc (100 µM), Glucosamine (GlcN, 5 mM) and 4-OH-tamoxifen (10 µM). The cell population growth in each well was evaluated using an Uptiblue-based-assay as the ratio of final fluorescence over the initial one (broken line) in the same well. Each determination corresponds to measurements performed in triplicate wells. Results are the mean±SEM of at least 7 independent experiments. Statistical analysis was performed using ANOVA followed by Dunnet’s post-test. *, P< 0.05; **, P< 0.01 when compared to untreated control; NS, not significant. (C) MCF-7 cells were cultured in presence of 1% FBS during 24 h, in the absence or presence of PUGNAc+GlcN and 4-OH-tamoxifen (10 µM) and then analysed by FACS after Annexin V-FITC/Propidium Iodide staining. Statistical analysis was performed using ANOVA followed by Tukey’s post-test *, P< 0.05; **, P< 0.01. Results correspond to the mean±SEM of at least 4 independent experiments.

Figure 2A shows that treatment with PUGNAc and/or GlcN had no significant effect on cell growth. 4-OH-Tamoxifen markedly reduced the growth of the cell population after 24 h of culture. Interestingly, the effect of 4-OH-Tamoxifen was partially reversed by the presence of either PUGNAc or GlcN, and completely reversed when adding both agents together. Similar results were observed after 48 h of culture with the different agents (Figure 2B). These results suggest that treatment with PUGNAc and GlcN protects MCF-7 cells form 4-OH-Tamoxifen-induced cell death.

We then studied more specifically the effects of 24h treatments on apoptosis using FACS analysis, after labelling the cells with Annexin V-FITC and propidium iodide. In absence of Tamoxifen, PUGNAc and GlcN had no significant effect on cell apoptosis. Tamoxifen induced a 2-fold increase in apoptosis, which was prevented by the presence of PUGNAc + GlcN (Figure 2C).

**Figure 3:**
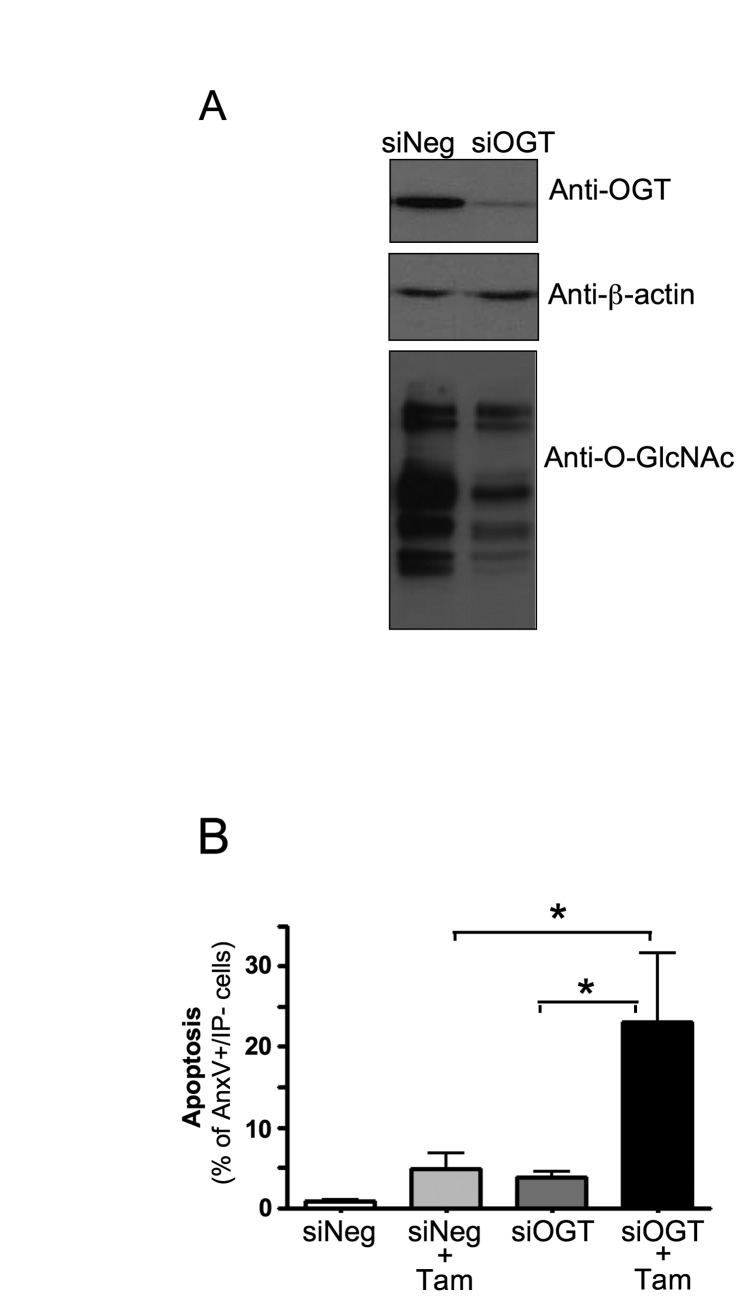
Inhibition of OGT expression sensitizes MCF-7 cells to tamoxifen-induced apoptosis. MCF-7 cells were transfected with control (siNEG) or anti-OGT siRNA (siOGT, sequence: TGGCATCGACCTCAAAGCA). (A) OGT protein expression and global O-GlcNAc levels were analysed 48h later by western-blot. (B) 48h after transfection with siRNA, cells were cultured for 24 hours in absence or presence of 4-OH-tamoxifen (10 µM) and then analysed by FACS after Annexin V-FITC/Propiduim Iodide staining. Statistical analysis was performed using ANOVA followed by Tukey’s post-test *, P< 0.05. Results correspond to the mean±SEM of 5 independent experiments.

To confirm the involvement of O-GlcNAc modifications in protection from 4OH-tamoxifen-induced cell death, we used siRNA to study the effect of inhibiting OGT expression on apoptosis. As shown in Figure 3A, the expression of OGT was inhibited in siOGT-transfected cells compared to control (siNeg-transfected) cells, which in turn resulted in a decrease in the level of O-GlcNAcylated proteins. Figure 3B shows that inhibition of OGT expression increased tamoxifen-induced cell death. Altogether, these results indicate that increased O-GlcNAcylation protects MCF-7 cells from tamoxifen-induced apoptosis, whereas inhibition of OGT sensitizes these cells to the effects of tamoxifen.

### O-GlcNAc-inducing treatments stimulate the PI-3 kinase/Akt pathway in MCF-7 cells

**Figure 4:**
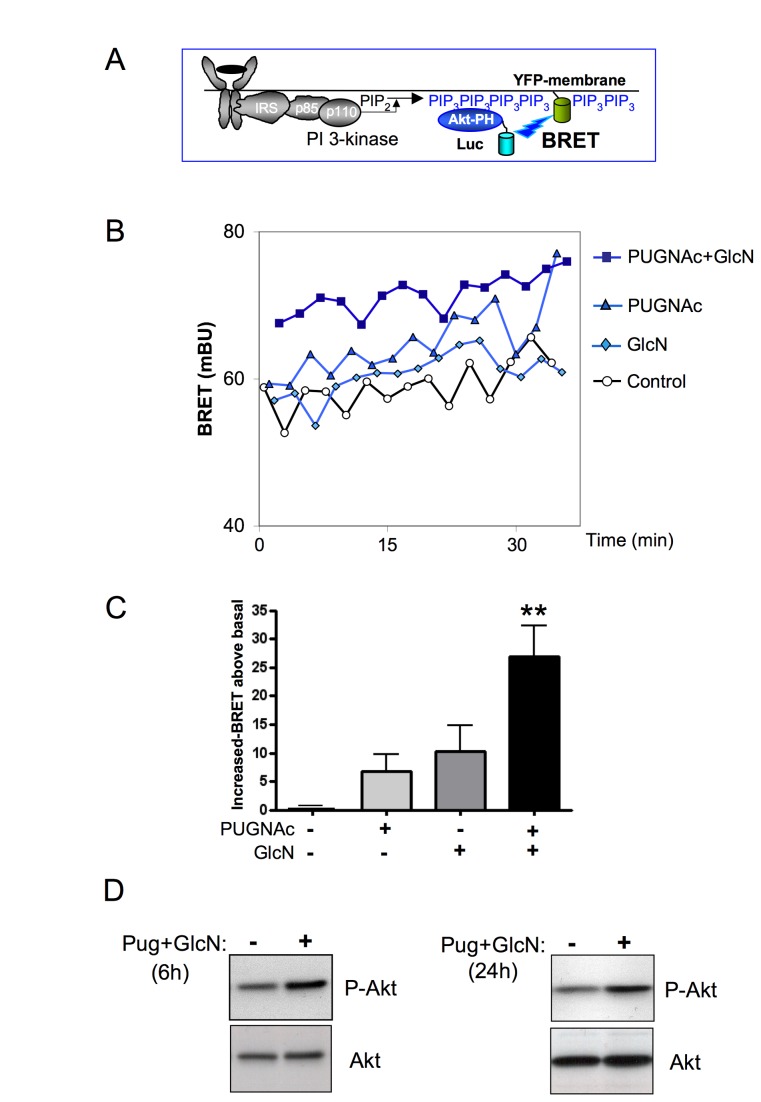
O-GlcNAc-inducing treatments stimulate the PI-3 kinase/Akt pathway in MCF-7 cells. (A) Principle of the BRET assay to measure PIP_3_ production in living cells. Activation of PI-3 kinase induces the phosphorylation of phosphatidyl-inositol 2 phosphate (PIP_2_) into phosphatidyl-inositol 3 phosphate (PIP_3_) and subsequent recruitment of Akt to the plasma membrane through its pleckstrin homology (PH) domain. To monitor the production of PIP_3_ induced by receptor activation, cells are co-transfected with cDNAs coding for the PH domain of Akt fused to luciferase (Luc-Akt-PH) and YFP fused to a membrane localization sequence. (B) 48 h after transfection, cells were pre-incubated for 6 h in presence of PUGNAc, GlcN or both. Cells were incubated for 10 min with coelenterazine, then light acquisition at 480 nm and 530 nm started. A typical experiment is shown. (C) PUGNAc and GlcNAc-induced BRET (BRET above basal at the plateau). Results are the means ± S.E.M. of 4 to 8 independent experiments. Statistical analysis was performed using ANOVA followed by Dunnet’s post-test. **, P< 0.01 when compared to untreated control. (D) Effect of O-GlcNAcylation-inducing treatment on Akt phosphorylation. MCF-7 cells were incubated in the absence or presence of PUGNAc+GlcN for 6h or 24 h and lysed. The phosphorylation of Akt was evaluated by western-blot using anti-phospho-S473-Akt antibody.

The PI-3 kinase/Akt pathway is known as a major anti-apoptotic pathway in MCF-7 cells. To determine whether O-GlcNAcylation-inducing treatments may affect this pathway, we used our previously developed BRET-based assay to monitor PIP_3_ production in intact living cells [25]. In this assay, cells are co-transfected with cDNA coding for the PH domain of Akt fused to *Renilla* Luciferase and YFP targeted to the plasma membrane (Figure 4A). Cells were incubated with PUGNAc, GlcN or both for 6h prior to BRET assay. Whereas treatment with PUGNAc or GlcN alone had only modest effects on PIP3 production, a significant increase was observed when both agents were added together (Figure 4B, C). In agreement with this result, we observed that PUGNAc+GlcN treatment for 6 or 24h resulted in increased Akt phosphorylation in MCF-7 cells (Figure 4D).

### Protection of 4-OH-Tamoxifen-induced cell death by O-GlcNAc-inducing treatments is independent of PI-3 kinase/Akt pathway

**Figure 5:**
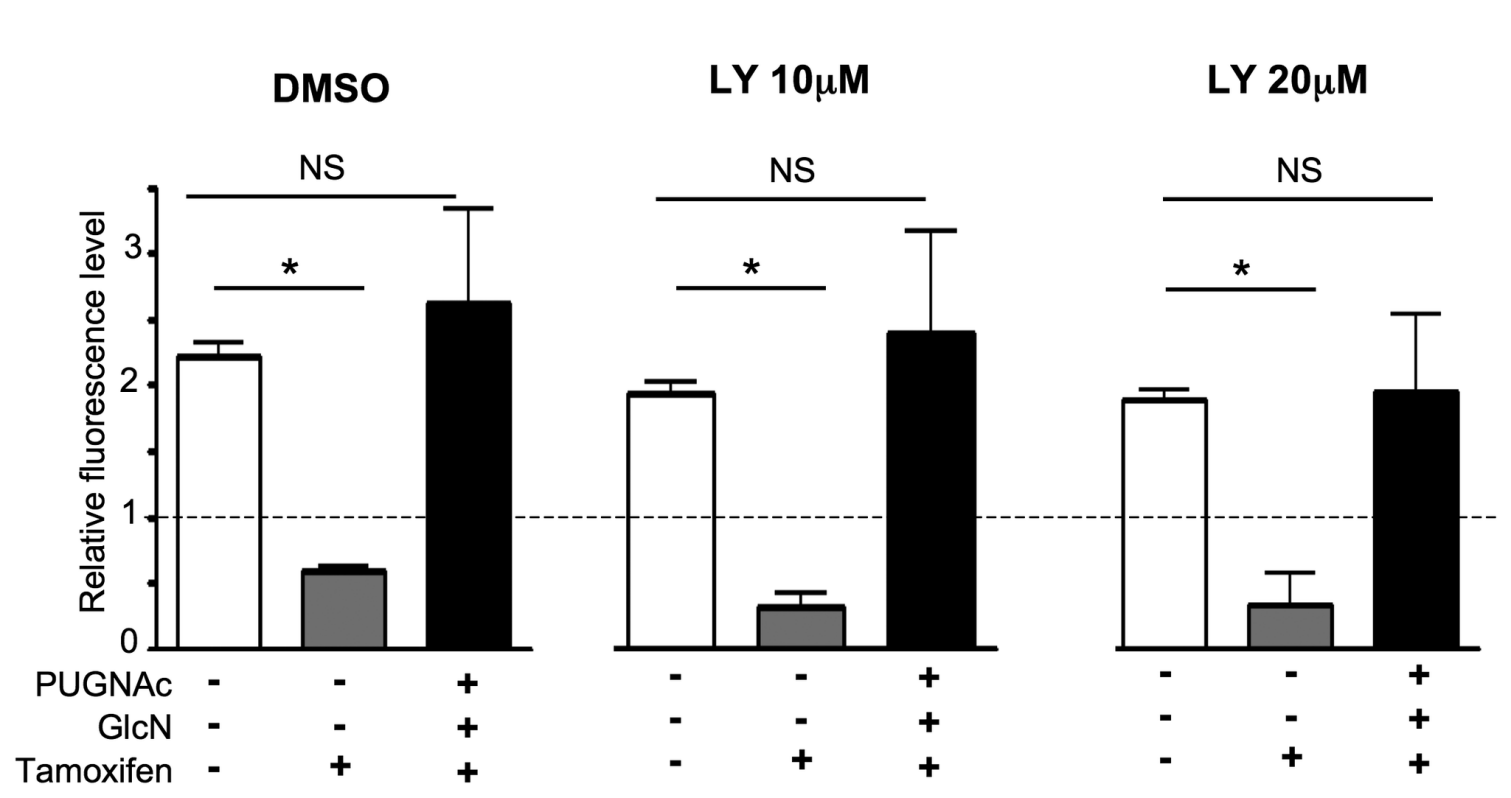
Protection of MCF-7 cells from 4-OH-tamoxifen-induced cell death is not abrogated by inhibition of the PI-3 kinase/Akt pathway. MCF-7 cells were cultured in presence of 1% FBS during 24 hours in the absence or presence PUGNAc (100 μM), Glucosamine (GlcN, 5 mM), 4-OH-tamoxifen (10 μM) and either DMSO (vehicle) or the PI-3 kinase inhibitor LY294002 (LY) at a concentration of 10 or 20 µM. The growth of the cell population in each well was evaluated using Uptiblue-based-assay as the ratio of final fluorescence over the initial one (broken line) in the same well. Each determination corresponds to measurements performed in triplicate wells. Results are the mean±SEM of 3 independent experiments. Statistical analysis was performed using ANOVA followed by Dunnet’s post-test. *, P< 0.05 when compared to corresponding untreated control; NS, not significant.

To determine whether PUGNAc+GlcN protective effects against 4-OH-Tamoxifen-induced cell death were mediated by their stimulatory effect on PI-3 kinase/Akt pathway, we evaluated the effects of these treatments in presence of LY294002, a PI-3 kinase inhibitor. We observed that LY294002, which markedly inhibited Akt phosphorylation in these conditions (Figure S2), did not impair PUGNAc+GlcN rescue from 4-OH-Tamoxifen-induced cell death (Figure 5). This result strongly suggested that the protective effect of O-GlcNAcylation-inducing treatment is not mediated by the PI-3 kinase pathway.

**Figure 6:**
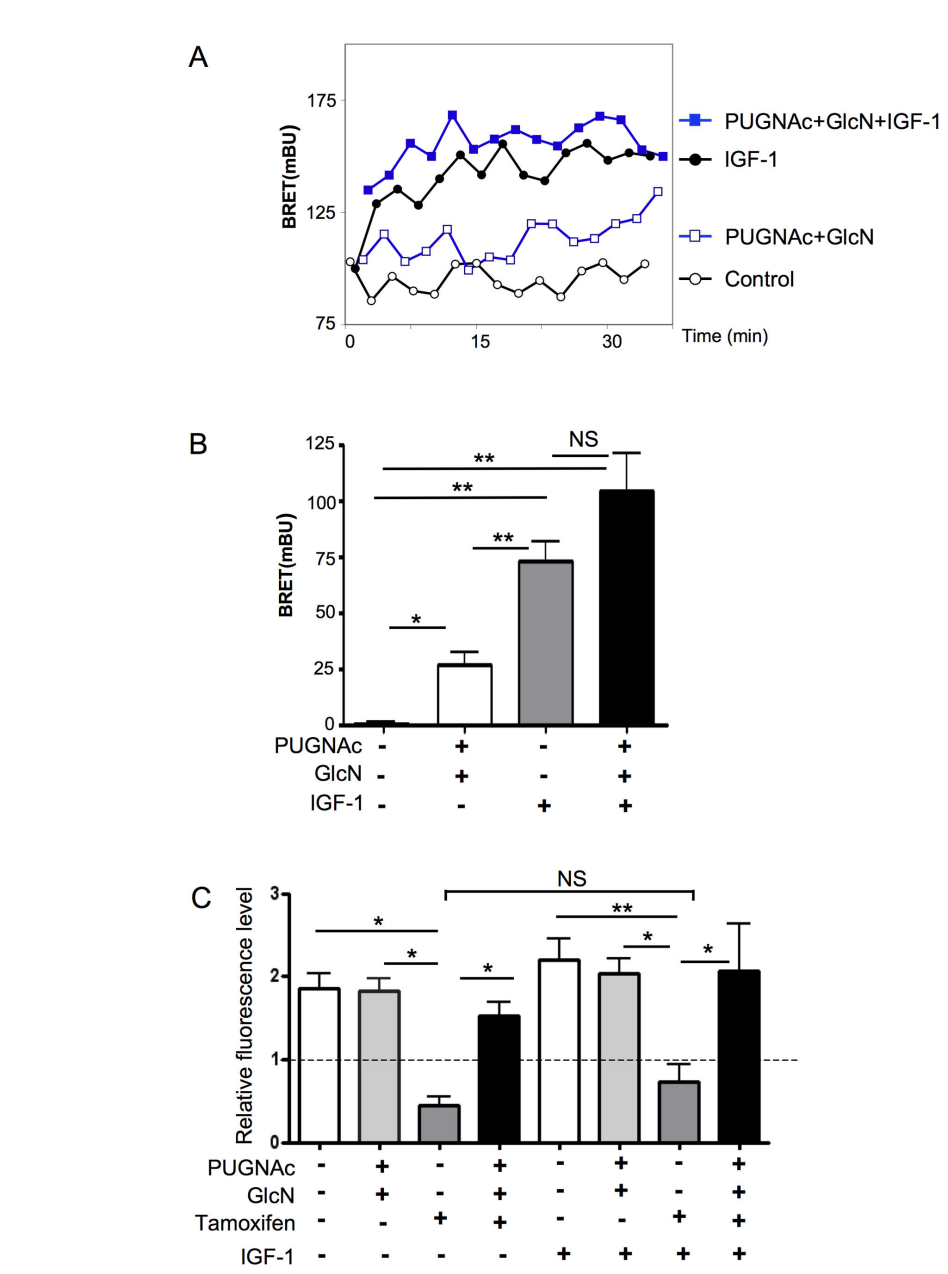
IGF-1 treatment does not protect MCF-7 cells from 4OH-tamoxifen-induced cell death. (A) MCF-7 cells were co-transfected with cDNAs coding for the PH domain of Akt fused to luciferase (Luc-Akt-PH) and YFP fused to a membrane localization sequence. 48 h after transfection, cells were pre-incubated for 6 h in the absence or presence of PUGNAc+GlcN. Cells were incubated for 10 min with coelenterazine and then stimulated with 100 nM IGF-1. Light acquisition at 480 nm and 530 nm started immediately after IGF-1 addition. A typical experiment is shown. (B) PUGNAc+GlcNAc and IGF-1-induced BRET (BRET above basal at the plateau). Results are the means ± S.E.M. of at least 5 independent experiments. Statistical analysis was performed using ANOVA followed by Tukey’s post-test. *, P< 0.05; **, P< 0.01; NS, not significant. (C) MCF-7 cells were cultured in presence of 1% FBS during 24 h in the absence or presence PUGNAc (100 µM), Glucosamine (GlcN, 5 mM), 4-OH-tamoxifen (10 µM) and IGF-1 (100 nM). Cell population growth in each well was evaluated using an Uptiblue-based-assay as the ratio of final fluorescence over the initial one (broken line) in the same well. Each determination corresponds to measurements performed in triplicate wells. Results are the mean±SEM of 5 independent experiments. Statistical analysis was performed using ANOVA followed by Tukey’s post-test. *, P< 0.05; **, P< 0.01; NS, not significant.

Independence of the PI-3kinase pathway was confirmed by evaluating the effect of IGF-1. As previously demonstrated [25], IGF-1 markedly stimulated PIP_3_ production in these cells (Figure 6A, B). Although IGF-1 had a much higher effect on PIP_3_ production than PUGNAc+GlcN (Figure 6B), no significant rescue from 4-OH-Tamoxifen-induced cell death was observed (Figure 6C). In contrast, the protective effect of PUGNAc+GlcN against tamoxifen induced cell death was still observed, and was similar in absence and presence of IGF1.

Therefore, although these experiments reveal a novel and highly interesting effect of O-GlcNAcylation-inducing treatments on the PI-3 kinase/Akt pathway, protection against 4-OH-Tamoxifen-induced cell death by these treatments appears to be independent of this pathway.

### ERα expression is inhibited by increased O-GlcNAcylation in MCF-7 cells

**Figure 7:**
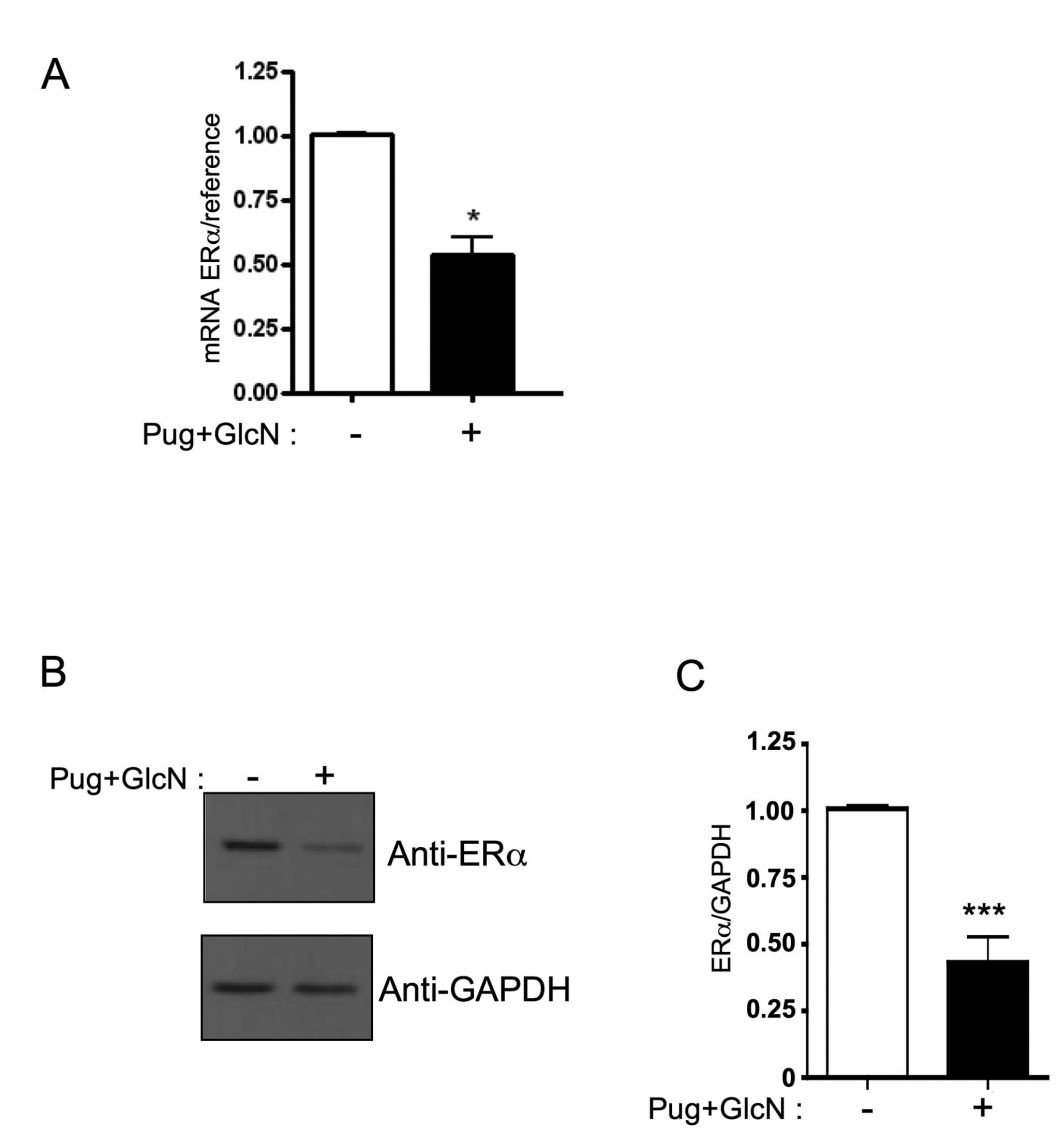
Inhibition of ERα expression level by O-GlcNAcylation-inducing treatments. Cells were cultured for 24 h in the absence or presence of PUGNAc+GlcN. (A) RNA were extracted and the expression of ERα mRNA was evaluated by RT-qPCR. (B) Cells were lysed and the expression of ERα protein was analysed by western-blot. GAPDH expression level was used as loading control. (C) ERα/GAPDH signals quantified by densitometric analysis of the autoradiograms of western-blots from 6 independent experiments. Statistical analysis was performed using unpaired t test. *, P< 0.05; ***, P< 0.001.

**Figure 8:**
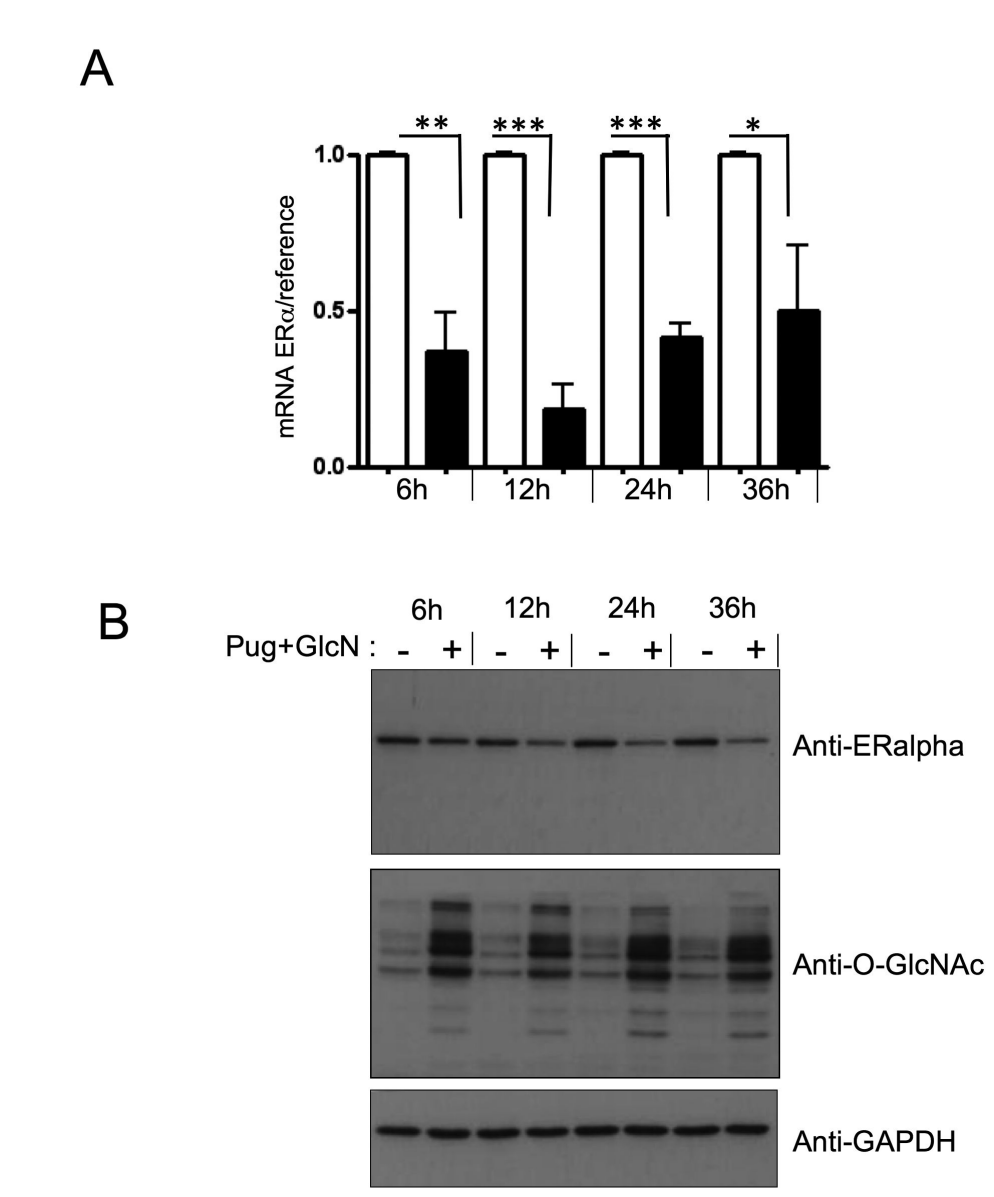
Time-course of inhibition of ERα expression by O-GlcNAcylation-inducing treatments. Cells were cultured in absence or presence of PUGNAc+GlcN for 6, 12, 24 and 36 h. (A) RNA was extracted and the expression of ERα mRNA was evaluated by RT-qPCR. Statistical analysis was performed using unpaired *t* test. *, P< 0.05; **, P< 0.01; ***, P< 0.001. Results correspond to the mean±SEM of at least 4 independent experiments. (B) Cells were lysed at the indicated times and the expression of ERα protein was analysed by western-blot. GAPDH expression level was used as loading control. The effect of PUGNAc+GlcN treatment on O-GlcNAcylation of proteins was controlled using anti-O-GlcNAc antibody. Results are representative of at least 3 independent experiments.

Tamoxifen effects on breast cancer are largely mediated by its antagonistic action against the estrogen receptor. A number of evidences indicate that the breast cancer cell sensitivity to tamoxifen depends on the expression level of the estrogen receptor ERα [33,34]. Low expression level of *ESR1* gene, which codes for ERα, is an important determinant of tamoxifen resistance in ER-positive breast tumors [35]. We hypothesised that treatment with O-GlcNAcylation-inducing agents may reduce 4-OH-Tamoxifen-induced cell death through inhibition of ERα. Therefore, we evaluated the effect of PUGNAc+GlcN on the expression of ERα in MCF-7 cells by RT-qPCR and western-blotting. As shown in Figure 7, 24h treatment of MCF-7 cells with PUGNAc+GlcN markedly reduced ERα expression at both the mRNA and protein levels. Time-course experiments (Figure 8) indicated that inhibition of ERα expression could already be detected at the mRNA level 6h after of treatment with PUGNAc+GlcN (Figure 8A), and was readily detected at the protein level after 12 h of treatment (Figure 8B).

**Figure 9:**
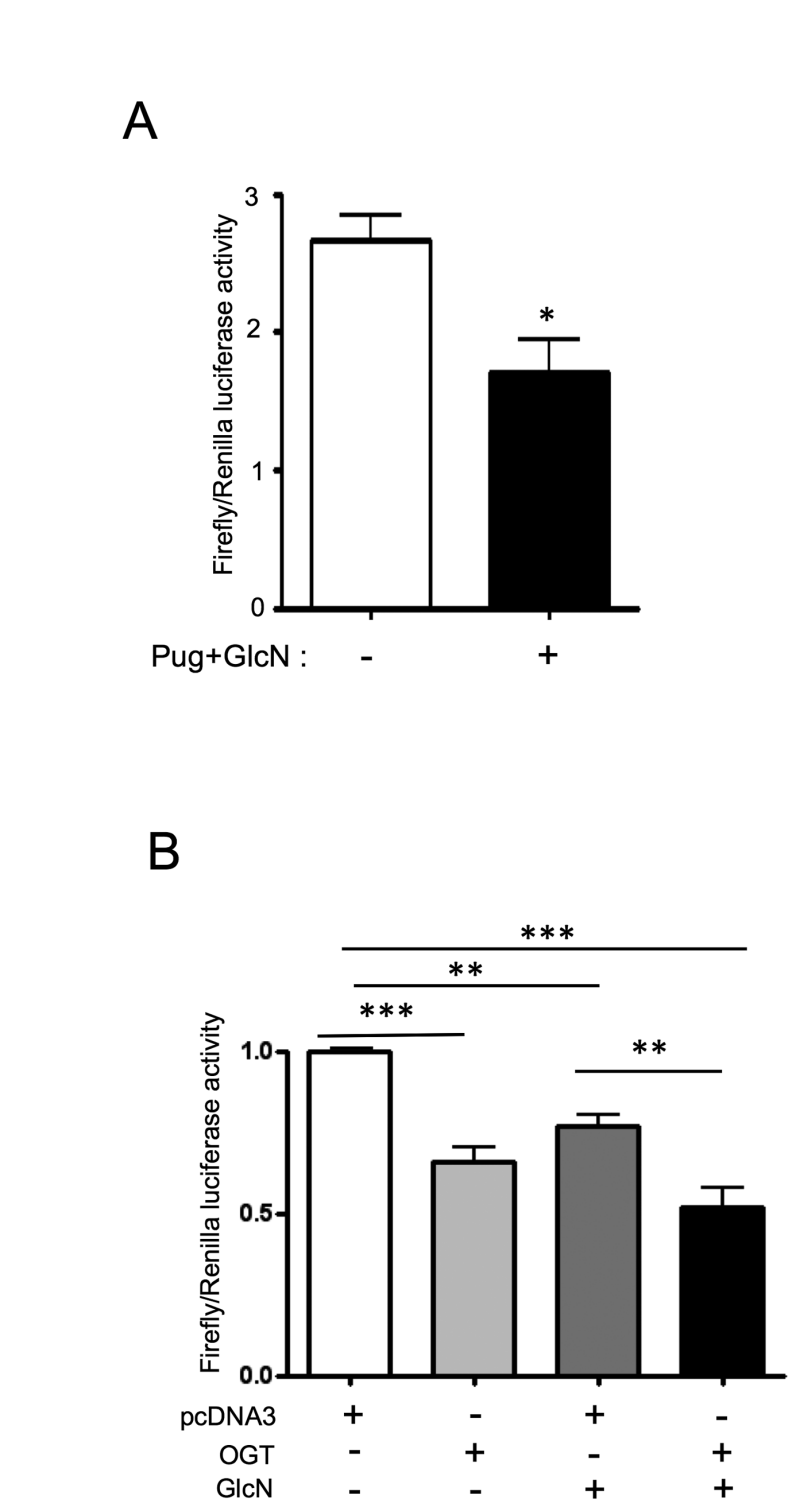
Inhibition of ESR1 promoter activity by O-GlcNAcylation-inducing treatments. (A) MCF-7 cells were co-transfected with *ESR1* promoter-Firefly luciferase reporter gene (*ESR1*-luc) and *Renilla* luciferase cDNAs. 12 h after transfection, cells were treated with PUGNAc+GlcN for 24 h and then lysed for determination of Firefly and Renilla luciferase activities. Each determination was performed in triplicate. Results are mean±SEM of three independent experiments. Statistical analysis was performed using unpaired *t* test ; *, P< 0.05. (B) Cells were transfected as in A, but the *ESR1*-Luc and *Renilla* luciferase plasmids were co-transfected with either pcDNA3.1 control plasmid or OGT expression vector. Cells were then treated with GlcN for 24 h and then lysed for determination of Firefly and Renilla luciferase activities. Each determination was performed in triplicate. Statistical analysis was performed using ANOVA followed by Tukey’s post-test **, P< 0.01; ***, P< 0.001. Results correspond to the mean±SEM of 4 independent experiments.

To determine whether O-GlcNAcylation-inducing treatments affect ERα expression through a transcriptional mechanism, we transfected MCF-7 cells with a reporter gene constituting of the firefly luciferase coding sequence under the control of *ESR1* promoter. Twelve hours after transfection, cells were treated with PUGNAc+GlcN for 24h, and then lysed for luciferase activity measurements. As shown in Figure 9A, PUGNAc+GlcN treatment markedly reduced the expression of *ESR1* promoter reporter gene. To further confirm the role of protein O-GlcNAcylation in the regulation of *ESR1* promoter, a luciferase reporter gene assay was performed after over-expression of OGT. Transfection with OGT cDNA increased OGT expression and protein O-GlcNAcylation in MCF-7 cells (Figure S3). Figure 9B shows that the *ESR1-Luc* reporter gene expression was significantly reduced when OGT cDNA was co-transfected with the reporter gene. This inhibitory effect was accentuated by glucosamine, which provides UDP-GlcNAc to OGT. We also evaluated the effect of PUGNAc+GlcN on ERα expression in presence of tamoxifen. The inhibitory effect of PUGNAc+GlcN on *ESR1* promoter activity was still observed when cells were cultured in presence of tamoxifen (Figure S4A). At the mRNA level, tamoxifen alone tended to decrease the expression of ERα As a consequence, in presence of tamoxifen, although PUGNAc+GlcN further decreased ERα mRNA level, the difference between cells treated or not with PUGNAc+GlcN was not significant. However, it should be noted that whereas the decrease in mRNA level induced by tamoxifen alone was not significant when compared to untreated control cells, this effect became significant in presence of PUGNAc+GlcN, indicating that PUGNAc+GlcN favours inhibition of ERα mRNA expression, even in presence of tamoxifen (Figure S4B). At the protein level, tamoxifen increased ERα, in agreement with previous data showing that tamoxifen increase ERα protein expression through a post-transcriptional mechanism [36], presumably by protecting ERα from proteasomal degradation [37,38]. However, despite tamoxifen-induced increase in ERα protein level, PUGNAc+GlcN treatment was still capable of significantly decreasing ERα protein expression (Figure S4C and D). This suggests that in presence of tamoxifen, under PUGNAc+GlcN conditions, less ERα protein is available to transmit tamoxifen effects compared to the minus PUGNAc+GlcN condition.

**Figure 10:**
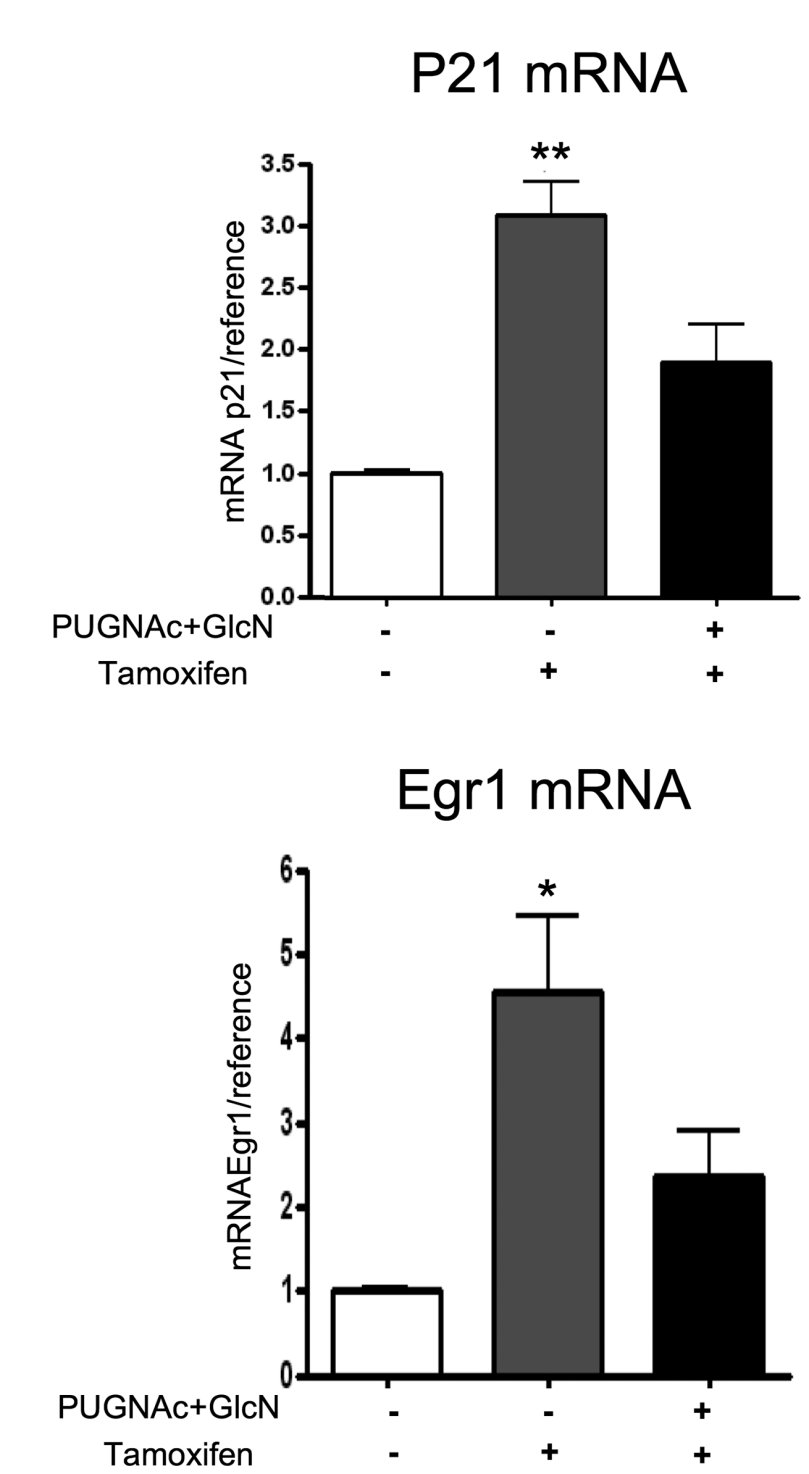
PUGNAc+GlcNAc treatment inhibited the effect of 4-OH-tamoxifen on *p21* and *Egr1* gene expression. Cells were cultured for 24h in absence or presence of 4-OH tamoxifen or in presence of 4-OH-Tamoxifen and PUGNAc+GlcN. (A) RNA were extracted and the level of p21 and Egr1 mRNA was evaluated by RT-qPCR and normalised using Cyclophilin A expression levels. Results are the mean ±SEM of 3 independent experiments. Statistical analysis was performed by ANOVA followed by Dunnet’s post-test. *, P< 0.05; **, P< 0.01 when compared to untreated control.

To determine whether O-GlcNAc-induced inhibition of ERα expression was associated with an inhibition of tamoxifen effect on gene expression, we studied the mRNA expression of genes coding for p21 and Egr1 by RT-qPCR. p21 and Egr1 negatively regulate cell proliferation and were previously shown to be activated by tamoxifen in MCF-7 cells [39,40]. We observed PUGNAc+GlcN reduced the stimulatory effect of tamoxifen on the expression of these genes (Figure 10).

Altogether, these data suggest that O-GlcNAcylation-inducing treatments inhibited ERα expression, and this inhibition was associated with a reduction of tamoxifen effect on key genes involved in regulation of cell proliferation.

## Discussion

O-GlcNAcylation has been implicated as an important determinant in cancer cell growth and migration [16–21]. However, its role in the sensitivity to anti-cancer therapy was not investigated in these studies. In the present work, we demonstrate that increasing O-GlcNAcylation results in important protective effects against tamoxifen-induced cell death in the MCF-7 cell line.

In MCF-7 cells, increased activity of the PI-3 kinase/Akt pathway has been previously shown to participate in resistance to various chemotherapeutic agents, including tamoxifen [41]. To evaluate the potential involvement of this pathway in the protective effects of O-GlcNAc-inducing treatments, we used our previously developed BRET-based assay to assess PIP_3_ level in living cells. We observed that PUGNAc+GlcN treatment significantly increased PIP_3_ production in MCF-7 cells and was associated with an increase in Akt phosphorylation. Interestingly, recent work also suggested a link between PI-3 kinase activity and OGT expression level in cancer cells [42]. The mechanism by which O-GlcNAc increases PI-3K/Akt pathway activity in MCF-7 cells remains elusive. Whereas O-GlcNAcylation of the p85 subunit [43] of PI-3K has been previously described, its consequences on its activity have not been studied. Moreover, whereas O-GlcNAcylation of Akt has been largely described [5,9,44,45], this modification is rather associated with a decrease in its phosphorylation. PIP_3_ level at the plasma membrane depends on the balance between PIP_2_ phosphorylation by PI-3 kinase activity and PIP_3_ dephosphorylation by the lipid-phosphatase PTEN. Clearly, the effect of O-GlcNAc treatment on PI-3 kinase and PTEN expression and/or activity in MCF-7 cells deserves further investigations.

Whatever the mechanism involved in O-GlcNAc-induced increase in PI-3-kinase/Akt pathway in these cells, it does not appear to play a significant role in the protective effect of O-GlcNAc against tamoxifen-induced cell death. Indeed, pharmacological inhibition of the PI-3 kinase/Akt pathway using LY294002 did not impair the protective effects of PUGNAc+GlcN treatments on 4-OH-tamoxifen induced cell death. Moreover, using IGF1, we demonstrated that activation of PI-3 kinase is not sufficient to rescue MCF-7 cells from tamoxifen effects, whereas PUGNAc+GlcNAc treatment was still efficient under these conditions. This prompted us to investigate whether O-GlcNAcylation, rather than acting through a general anti-apoptotic signalling pathway, may affect a protein more specifically involved in tamoxifen action.

Tamoxifen exerts its effects on estrogen-receptor positive cells by binding to ERα. ERα expression is both necessary and sufficient to predict the responsiveness to anti-estrogen in a high proportion of breast tumors, and low expression level is generally associated with a poor prognosis [33–35,46,47].

We observed that O-GlcNAcylation-inducing treatment results in inhibition of ERα expression, suggesting a potential mechanism for decreased tamoxifen effect. Inhibition of ERα mRNA expression could be detected after only 6 h of treatment, and luciferase reporter gene assay indicated that PUGNAc+GlcN treatments inhibit the activity of the ESR1 promoter reporter gene. This suggests that the inhibition of ERα expression occurred at least in part through a transcriptional repression mechanism. This inhibition appeared to be independent of the effect of O-GlcNAc on PI-3 kinase/Akt pathway, because treatment of MCF-7 cells with LY294002 did not impair inhibition of ERα expression by PUGNAc+GlcN treatment (data not shown).

Positive and negative regulation of gene expression by post-translational modification of transcription factors has been largely documented [8,48,49]. Interestingly, Sp1, one the first transcription factors identified to be modified by O-GlcNAcylation [50], is known to bind to and positively regulate the *ESR1* promoter activity [51]. Indeed, Sp1 appears to participate in a transcription complex (comprised of USF1 and ERα itself) that regulates the expression of *ESR1*. O-GlcNAcylation of Sp1 has been largely associated with decreased transcriptional activity on various promoters, generally through dissociation of its interaction with transcription factors or co-activators [52–58]. Therefore, is would be of interest to determine whether the effect of PUGNAc+GlcN in MCF-7 cells is associated with dissociation of this complex.

Expression of the ERα, a good prognostic factor in breast cancer, is associated with higher levels of p21 proteins. Tamoxifen is known to modulate the expression of genes involved in cell proliferation and cell death. Increased expression of the cell cycle regulator p21^WAF1/CIP1^ is believed to play a role in cell cycle arrest upon treatment with tamoxifen and other anti-estrogen drugs [39,59]. Moreover, down-regulation of p21^WAF1/CIP1^ using antisense RNA abrogates anti-estrogen-mediated cell cycle arrest in MCF-7 cells [39]. Similarly, Egr1 is deleted in ER-negative human breast carcinoma, which is suggested to contribute to the poor prognosis of ER-negative versus ER-positive breast carcinomas [60]. Decreased Egr1 expression in human, mouse and rat mammary cells and tissues correlated with tumor formation, and tamoxifen treatment was shown to restore its expression in rat mammary tumors [40]. Moreover, in the ER-positive breast cancer cells MDA-MB-361, tamoxifen-induced increase in Egr1 expression resulted in activation p21^WAF1/CIP1^ promoter and increased transcription of *p21* ^WAF1/CIP1^ gene, providing a link between *Egr1* transcriptional activity, *p21* expression and tamoxifen-induced cell cycle arrest [61]. Our results indicated that O-GlcNAcylation-inducing treatment reduced the stimulatory effect of tamoxifen on *Egr1* and *p21* expression, providing a potential mechanism for the protective effects of O-GlcNAc on tamoxifen-induced inhibition of cell growth. However, the effect tamoxifen on *Egr1* and *p21* gene expression is only partially inhibited by O-GlcNAc-inducing treatments. This may in part reflect the fact that O-GlcNAc-induced inhibition of ERα expression is only partial (Figures 7 and 8), and the remaining receptors could be responsible for the residual effect of tamoxifen.

In summary, we have observed that increased O-GlcNAcylation in MCF-7 cells have inhibitory effects on tamoxifen induced cell death. Although O-GlcNAc-inducing treatments stimulate the PI-3 kinase/Akt pathway through an unknown mechanism, protection from tamoxifen-induced cell death appear to be independent of this pathway. Moreover, we observed that increased O-GlcNAcylation reduces the expression of ERα, one of the main determinants of tamoxifen sensitivity. However, several laboratories have shown that tamoxifen can have ERα-independent anti-proliferative and pro-apotptotic effects [62–64]. In the present study, it cannot be ruled out that O-GlcNAcylation may also protect MCF-7 cells from ERα-independent effects of tamoxifen. Therefore, additional work will be required to determine to what extent reduction in ERα expression is responsible for reduced tamoxifen effect upon O-GlcNAcylation-inducing treatment. Overall, although no firm conclusion can be drawn concerning the mechanism(s) by which O-GlcNcylation protects MCF-7 cells from tamoxifen effects, our results suggest that targeting the O-GlcNAc pathway might be an interesting therapeutic approach for sensitisation of anti-estrogen resistant breast tumors.

## Supporting Information

Figure S1 Effect of PUGNAc and Glucosamine on O-GlcNAc level in MCF-7 cells treated or not with 4-OH-tamoxifen. MCF-7 cells cultured in absence (Ct) or presence of PUGNAc (P), glucosamine (G) or PUGNAc+glucosamine (PG), were treated or not with of 4-OH-tamoxifen (10 µM). After 24 h of treatment, cells were lysed and protein O-GlcNAcylation level was evaluated by western-blotting. GAPDH expression level was used as a loading control.
(TIF)

Figure S2 Effect of LY294002 on Akt phosphorylation in MCF-7 cells. MCF-7 cells treated with 10 or 20 µM LY294002 (LY) or vehicle (DMSO) were cultured during 24 h in absence or presence PUGNAc+GlcN and/or tamoxifen. Cells were lysed and Akt phosphorylation level was evaluated by western-blotting using anti-phospho-S473-Akt antibody. As a control for specificity of PI-3 kinase inhibition, Erk phosphorylation (evaluated using anti-phospho-Erk1/2 antibody) was shown to be unaffected by LY294002 treatment in the same experiments.
(TIF)

Figure S3 Effect of OGT transfection on OGT expression and O-GlcNAcylation level in MCF-7 cells. MCF-7 cells were transfected with either pcDNA3 or OGT cDNA. 48 h after transfection, cells were lysed. OGT expression and O-GlcNAcylation level of proteins were evaluated by western-blotting. GAPDH expression level was used as a loading control.
(TIF)

Figure S4 Effect of PUGNAc+GlcN on *ESR1* promoter and ERα expression in presence of 4-OH-tamoxifen. (A) MCF-7 cells were co-transfected with *ESR1* promoter-Firefly luciferase reporter gene (*ESR1*-luc) and *Renilla* luciferase cDNAs. 12 hours after transfection, cells were treated with PUGNAc+GlcN in the absence or presence of 4-OH-tamoxifen for 24 h and then lysed for determination of Firefly and Renilla luciferase activities. Each determination was performed in triplicate. Results are mean±SEM of three independent experiments. Statistical analysis was performed using ANOVA followed by Tukey’s post-test. *, P< 0.05; **, P< 0.01; NS, not significant (B) Cells were cultured for 24 h in the absence or presence of PUGNAc+GlcN and 4-OH-tamoxifen. RNA was then extracted and the expression of ERα mRNA was evaluated by RT-qPCR. Results are the mean±SEM of 4 independent experiments. Statistical analysis was performed using ANOVA followed by Tukey’s post-test. *, P< 0.05; **, P< 0.01; NS, not significant. (C) Cells were lysed and the expression of ERα protein was analysed by western-blot. GAPDH expression level was used as loading control. (D) ERα/GAPDH signals quantified by densitometric analysis of the autoradiograms of western-blots from 6 independent experiments. Statistical analysis was performed using ANOVA followed by Tukey’s post-test. ***, P< 0.001.
(TIF)
