# Supplementary figures and images for: O-GlcNAcylation-Inducing Treatments Inhibit Estrogen Receptor α Expression and Confer Resistance to 4-OH-Tamoxifen in Human Breast Cancer-Derived MCF-7 Cells
Source: PLoS One. 2013 Jul 11;8(7):e69150. doi: 10.1371/journal.pone.0069150 (PMC3730543; doi:10.1371/journal.pone.0069150)

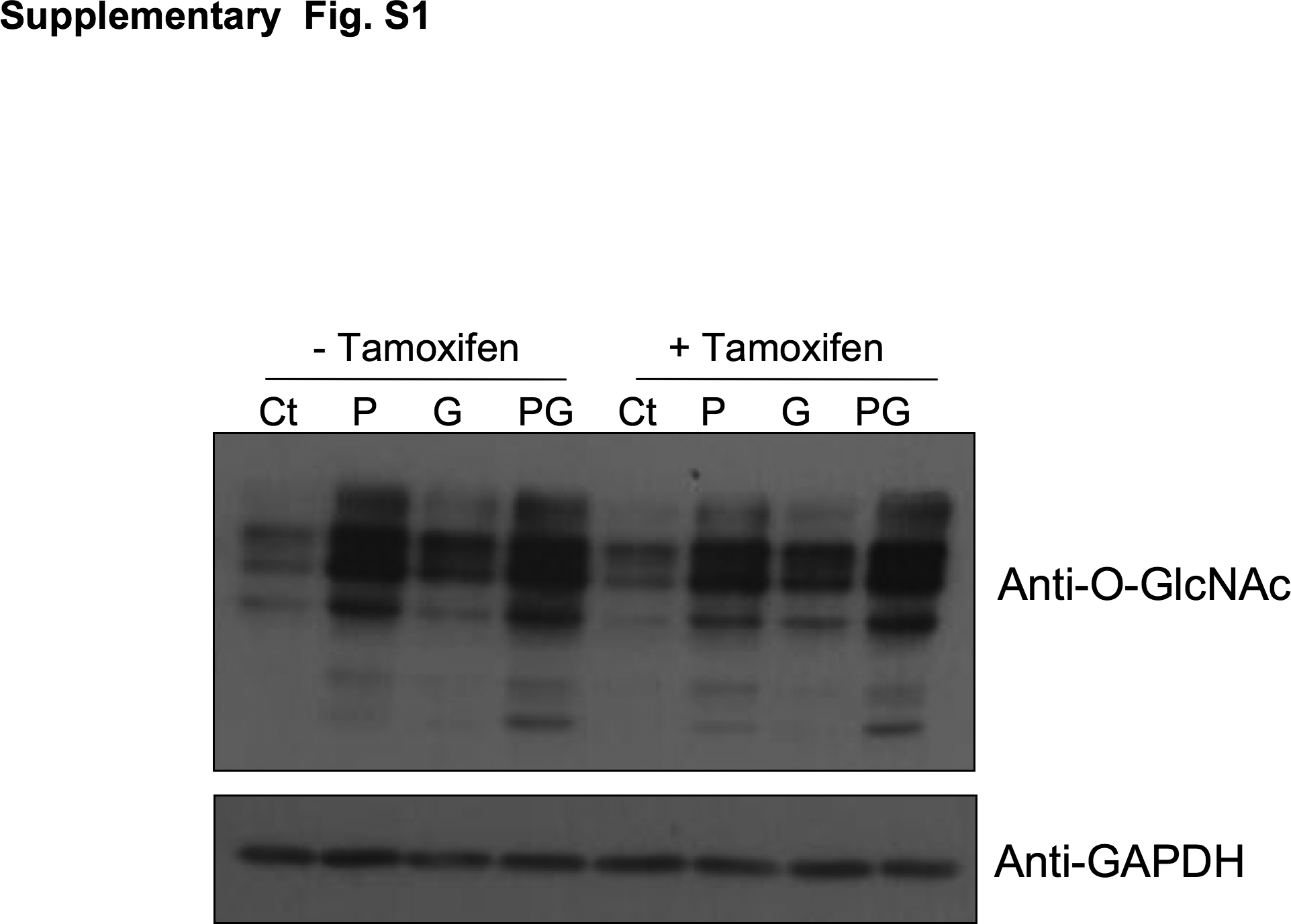

Supplement: Figure S1 — MCF-7 cells cultured in absence (Ct) or presence of PUGNAc (P), glucosamine (G) or PUGNAc+glucosamine (PG), were treated or not with of 4-OH-tamoxifen (10 µM). After 24 h of treatment, cells were lysed and protein O-GlcNAcylation level was evaluated by western-blotting. GAPDH expression level was used as a loading control. (TIF) [file pone.0069150.s001.tif]

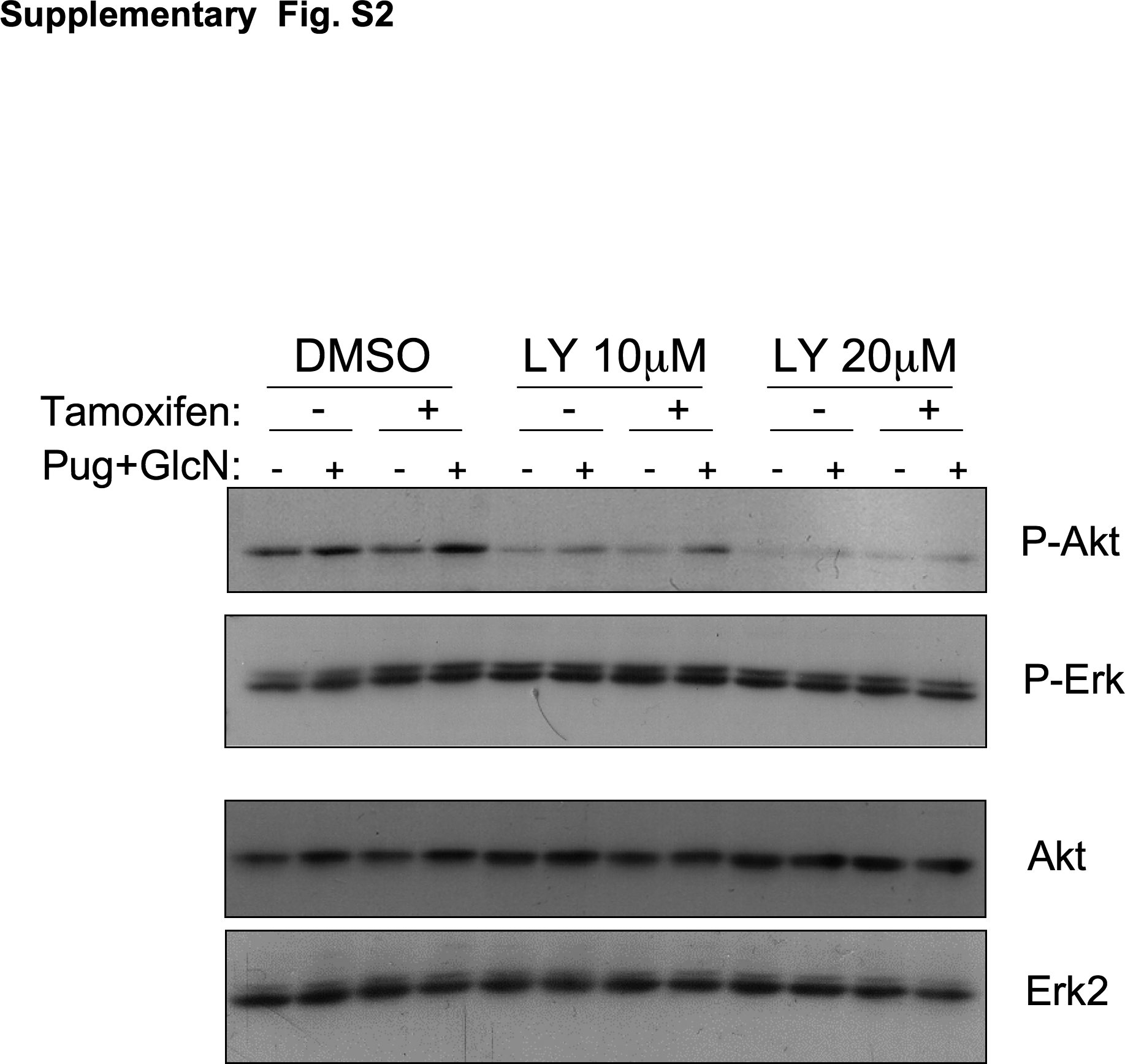

Supplement: Figure S2 — MCF-7 cells treated with 10 or 20 µM LY294002 (LY) or vehicle (DMSO) were cultured during 24 h in absence or presence PUGNAc+GlcN and/or tamoxifen. Cells were lysed and Akt phosphorylation level was evaluated by western-blotting using anti-phospho-S473-Akt antibody. As a control for specificity of PI-3 kinase inhibition, Erk phosphorylation (evaluated using anti-phospho-Erk1/2 antibody) was shown to be unaffected by LY294002 treatment in the same experiments. (TIF) [file pone.0069150.s002.tif]

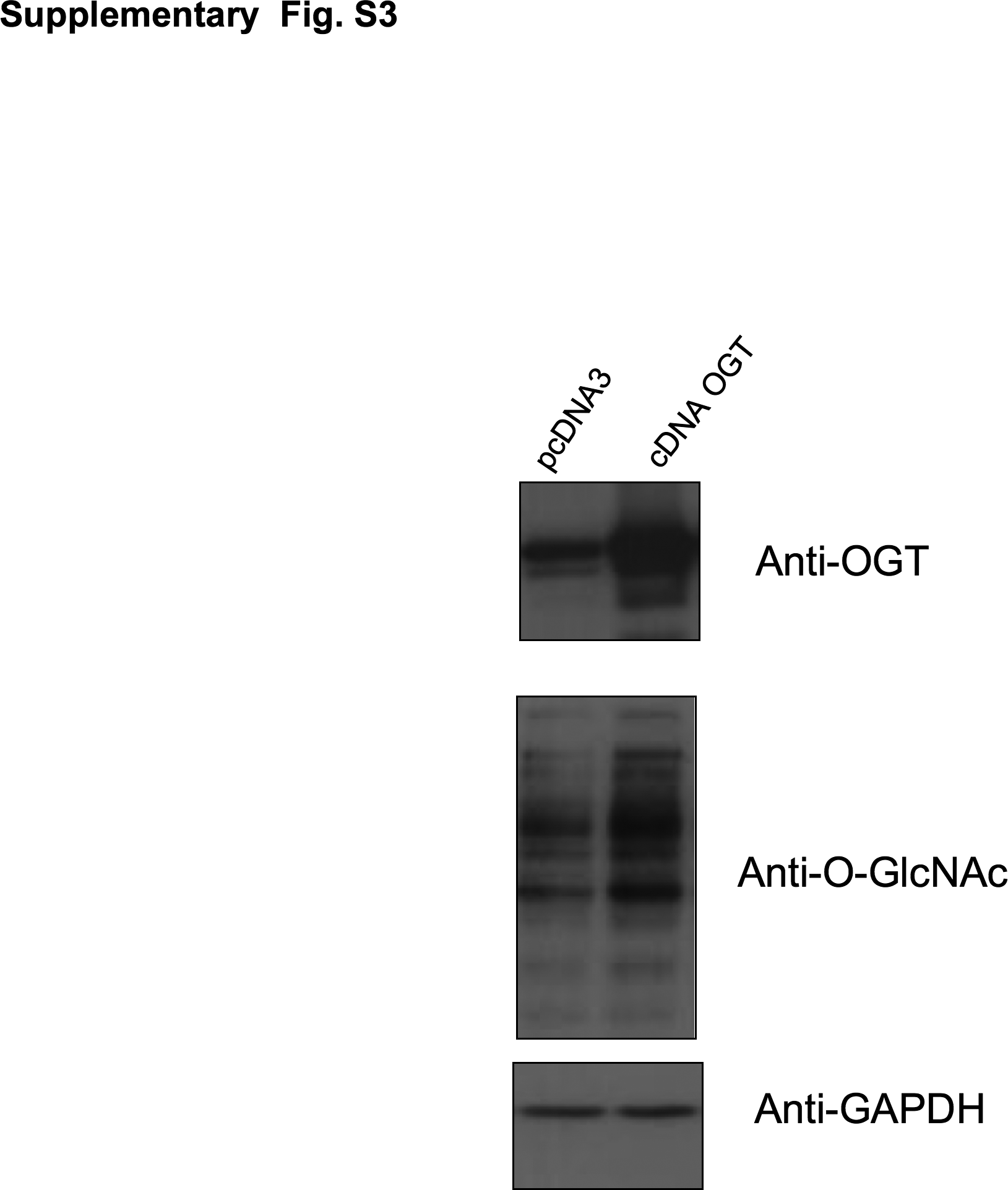

Supplement: Figure S3 — MCF-7 cells were transfected with either pcDNA3 or OGT cDNA. 48 h after transfection, cells were lysed. OGT expression and O-GlcNAcylation level of proteins were evaluated by western-blotting. GAPDH expression level was used as a loading control. (TIF) [file pone.0069150.s003.tif]

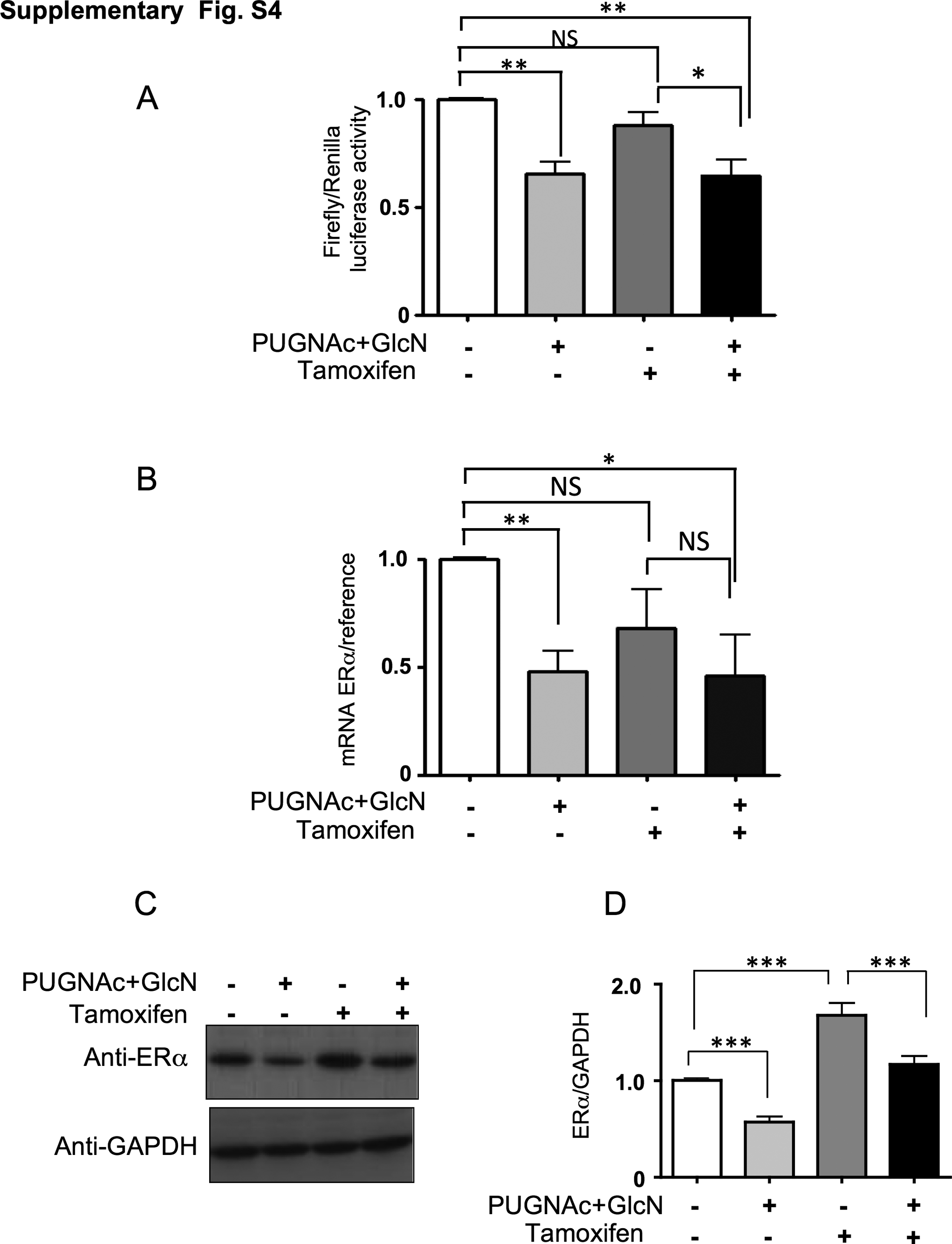

Supplement: Figure S4 — (A) MCF-7 cells were co-transfected with ESR1 promoter-Firefly luciferase reporter gene (ESR1-luc) and Renilla luciferase cDNAs. 12 hours after transfection, cells were treated with PUGNAc+GlcN in the absence or presence of 4-OH-tamoxifen for 24 h and then lysed for determination of Firefly and Renilla luciferase activities. Each determination was performed in triplicate. Results are mean±SEM of three independent experiments. Statistical analysis was performed using ANOVA followed by Tukey’s post-test. *, P< 0.05; **, P< 0.01; NS, not significant (B) Cells were cultured for 24 h in the absence or presence of PUGNAc+GlcN and 4-OH-tamoxifen. RNA was then extracted and the expression of ERα mRNA was evaluated by RT-qPCR. Results are the mean±SEM of 4 independent experiments. Statistical analysis was performed using ANOVA followed by Tukey’s post-test. *, P< 0.05; **, P< 0.01; NS, not significant. (C) Cells were lysed and the expression of ERα protein was analysed by western-blot. GAPDH expression level was used as loading control. (D) ERα/GAPDH signals quantified by densitometric analysis of the autoradiograms of western-blots from 6 independent experiments. Statistical analysis was performed using ANOVA followed by Tukey’s post-test. ***, P< 0.001. (TIF) [file pone.0069150.s004.tif]
